# CancerLivER: a database of liver cancer gene expression resources and biomarkers

**DOI:** 10.1093/database/baaa012

**Published:** 2020-03-07

**Authors:** Harpreet Kaur, Sherry Bhalla, Dilraj Kaur, Gajendra PS Raghava

**Affiliations:** 1 Bioinformatics Centre, CSIR-Institute of Microbial Technology, Sector -39A, Chandigarh-160036, India; 2 Department of Computational Biology, Indraprastha Institute of Information Technology, New Delhi-110020, India; 3 Centre for Systems Biology and Bioinformatics, Sector-25, Panjab University, Chandigarh-160036, India

**Keywords:** Resource, Liver Cancer, transcriptomics, Biomarkers, Expression, Datasets

## Abstract

Liver cancer is the fourth major lethal malignancy worldwide. To understand the development and progression of liver cancer, biomedical research generated a tremendous amount of transcriptomics and disease-specific biomarker data. However, dispersed information poses pragmatic hurdles to delineate the significant markers for the disease. Hence, a dedicated resource for liver cancer is required that integrates scattered multiple formatted datasets and information regarding disease-specific biomarkers. Liver Cancer Expression Resource (CancerLivER) is a database that maintains gene expression datasets of liver cancer along with the putative biomarkers defined for the same in the literature. It manages 115 datasets that include gene-expression profiles of 9611 samples. Each of incorporated datasets was manually curated to remove any artefact; subsequently, a standard and uniform pipeline according to the specific technique is employed for their processing. Additionally, it contains comprehensive information on 594 liver cancer biomarkers which include mainly 315 gene biomarkers or signatures and 178 protein- and 46 miRNA-based biomarkers. To explore the full potential of data on liver cancer, a web-based interactive platform was developed to perform search, browsing and analyses. Analysis tools were also integrated to explore and visualize the expression patterns of desired genes among different types of samples based on individual gene, GO ontology and pathways. Furthermore, a dataset matrix download facility was provided to facilitate the users for their extensive analysis to elucidate more robust disease-specific signatures. Eventually, CancerLivER is a comprehensive resource which is highly useful for the scientific community working in the field of liver cancer.**Availability**: CancerLivER can be accessed on the web at https://webs.iiitd.edu.in/raghava/cancerliver.

## Introduction

According to GLOBOCAN 2018, liver cancer is among the top five cancers with the highest mortality rate that accounts for 8.2% of the deaths caused by cancer. Nearly 841,000 new cases and 782,000 deaths of liver cancer have been estimated in 2018 worldwide (1). The major type of liver cancer is hepatocellular carcinoma (HCC) which accounts for nearly 75 to 85% of liver cancer cases. Another type of liver cancer is cancer in the bile duct (cholangiocarcinoma) which accounts for the 10 to 20% of total cases of liver cancer. Further, fibrolamellar carcinoma, hepatoblastoma, angiosarcoma and hemangiosarcoma are among the rare types of liver cancer. Fibrolamellar carcinoma tends to develop in adults 20–30 years old, and it is not usually associated with cirrhosis or infection with hepatitis B or C, while hepatoblastoma usually affects young children, i.e. children under 3 years. Angiosarcoma and hemangiosarcoma are the other rare types of liver cancers that start in the cell lining the blood vessels of the liver (2).

With the advancement in the field of genomics, enormous data have been generated to study the transcriptome expression of cancer samples to gain insights about the physiology of the disease. In the past, a wide range of high-throughput studies have been performed to identify cancer-specific biomarkers. The data generated from most of these high-throughput studies have been deposited over the time in various databases like Genomic Data Commons (GDC) data portal (3), International Cancer Genome Consortium (ICGC) Data Portal (4), The Cancer Genome Atlas (TCGA) (5) and Gene Expression Omnibus (GEO) (6). These databases primarily act as raw data repositories. Therefore, it is a formidable task to ascertain the biological significance from these data and make it available to users with an easy interface to analyse the data. This requires highly developed bioinformatics skills to annotate and generate appropriate data matrices to extract vital genes associated with the disease. Although GEO contains both raw and processed datasets, the processed datasets implement diverse processing and normalisation techniques. Therefore, the heterogeneity in the processed data poses major obstacles to compare various transcriptomics datasets. Thus, it is important to employ a uniform pipeline for the processing of a large number of raw datasets generated by a specific technique, i.e. Affymetrix or Illumina, to analyse them in a comprehensive manner to dissect more robust gene signatures for the disease.

In the recent past, there are a number of dedicated genomics, proteomics and peptidomics web resources and platforms designated for different types of cancers or disease condition like Colorectal Cancer Atlas (7), CRCRpred (8), IGDB.NSCLC (9), CancerPPD (10), Cancertope (11), HCMDB (12), StemMapper (13), CancerCSP (14), CancerPDF (15), CancerSPP (16), PhenoDis (17), RareLSD (18) and Clinical Genomic Database (CGD) (19) covering various disease conditions including colorectal cancer, non-small cell lung carcinoma, metastatic cancers, renal cell cancer, skin cancer and rare diseases.

There is an enormous generation of information and expression profiles of liver cancer patients which are deposited in various repositories and literature in different formats. Till date, there is no dedicated platform which maintains uniform datasets of liver cancer. Although, there is a resource Liverome (20), a curated database of liver cancer-related gene signatures which harbour information regarding signature genes associated with liver cancer from articles published up to the year 2010. It was not updated after its first publication in the year 2011. Besides, few of expression datasets for liver cancers are also available in resources like cBioPortal (21), BioXpress (22) and OncoMX (23). Among them, cBioPortal contains a total of eight datasets for liver cancer, three of which are of expression datasets, while five are of mutation datasets. BioXpress and OncoMX have taken the expression dataset from TCGA only. In addition, recently web portals such as HCCpred and CancerLSP are also developed for HCC prediction and the stage identification of liver cancer using transcriptomics and epigenomics data implementing machine learning algorithms, respectively (24, 25). This indicates that the information and datasets are widely scattered across different resources. Thus, there is a need to develop an integrated dedicated user-friendly public web resource or platform which catalogues the uniform data matrices for each specific type of expression profiling technique and the information regarding already identified/existing important markers for liver cancer.

To complement the existing database, here we present the liver cancer resource named as CancerLivER (https://webs.iiitd.edu.in/raghava/cancerliver), which provides annotated uniform matrices of published liver cancer expression profiles for each type of expression profiling techniques. Besides expression profiles, this resource also encompasses the information of various types of biomarkers or potential biomarker candidates for liver cancer mined from literature. This database is freely accessible to the research community to query and analyse liver cancer-related expression data and biomarkers.

## Material and methods

### Data collection

#### Gene-expression profile datasets

Systematic data searching was conducted for liver cancer expression profiles using the following keywords: ‘Liver cancer’, with customized criteria on study type ‘Expression profiling by array’, ‘Expression profiling by high throughput sequencing’, ‘Non-coding RNA profiling by array’ and ‘Non-coding RNA profiling by high throughput sequencing’ and criteria on organism ‘*Homo sapiens*’ from GEO (6) and GDC data portal (3). The extracted data are limited to human studies published before May 2018. A total of ~200 raw or supplementary datasets were initially downloaded using GEOquery package (26) and gdc-client from GEO and GDC data portal, respectively. Then, we manually curated these datasets to ensure that the data contains only expression data from human samples. Those datasets were excluded from the study where expression data from (i) cell lines and (ii) mice or rat. The number of datasets was reduced to ~160. Further, we exclude those datasets where SuperSeries are present and whose SubSeries were already included in the study; those datasets were also excluded from the study for which raw files were corrupted and where probe ID and HGNC Gene symbols were not available. Subsequently, only those datasets were included which have enough number of samples (at least two pairs, i.e. four samples). Eventually, the gene expression profiles of 115 datasets remained, including a total of 9611 samples that include cancerous, normal adjacent non-tumour and cirrhotic condition. In addition to expression profiles of samples, we also retrieved clinical information for the samples wherever available.

#### Processing of datasets

Each retrieved raw dataset (Supplementary Data) was subjected to a detailed curation process. For processing of Affymetrix gene expression datasets, the raw data (CEL files) were processed and normalized to RMA values implementing oligo package from Bioconductor (27) using a customized R pipeline. For processing of Agilent array data (both one-colour and two-colour), raw files were processed and normalized to A-values implementing Limma package (28, 29) from Bioconductor using a customized R-pipeline, in which at first a two-colour microarray background was corrected using ‘normexp’ with offset 0. For the processing of Illumina data, raw files were processed implementing Limma (28, 29) and lumi packages (30, 31) from Bioconductor (29) using a customized R pipeline. High-throughput datasets were manually curated using in-house scripts to make an appropriate matrix from different sample files for each dataset.

**Figure 1:**
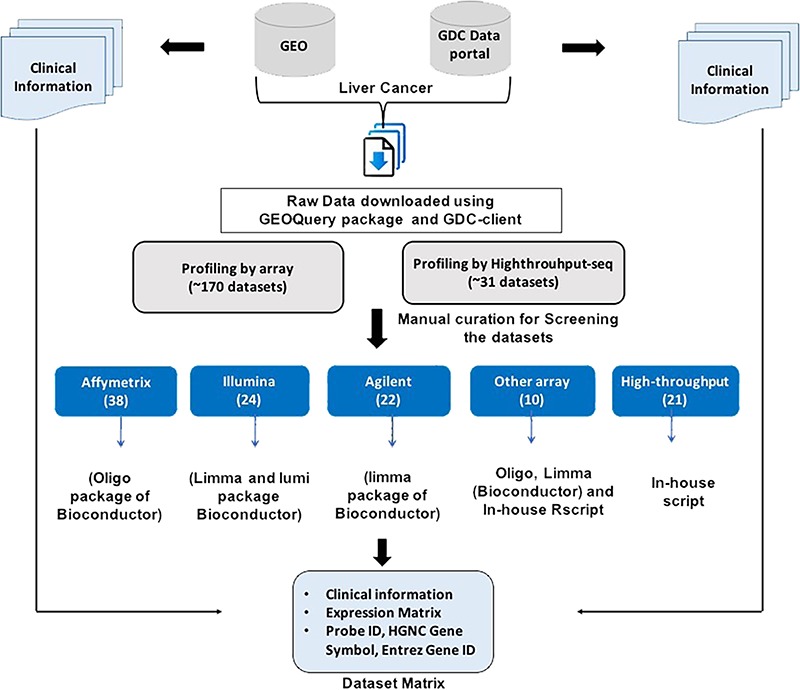
Workflow to generate Expression Dataset matrices maintained in CancerLivER.

The raw records for each dataset were manually inspected to extract relevant information including type, number, the source of samples and clinical information like age, gender, vital status and survival status. Further, each dataset carefully investigated to check the appropriate sample ID and type of sample and to remove any irrelevant error in the dataset file. In addition, HGNC Gene symbol and Entrez Gene ID were extracted from the respective Platform file (wherever available) and incorporated in the dataset matrix for each dataset. Eventually, a ready-to-use matrix for each dataset was prepared which consisted of clinical information (wherever available) and the expression profile of each gene/probe corresponding to each sample. Figure 1 represents the workflow to generate the final expression profile matrices.

#### Biomarker data

The data was manually collected and curated from published research articles. Only those candidate biomarkers were included in the resource, whose biomarker potential was experimentally determined. We queried PubMed to search for research articles regarding biomarkers for liver cancer. The query ‘(Liver cancer [Title/Abstract]) AND Biomarkers [Title/Abstract]’ was used to retrieve the articles relevant to liver cancer biomarkers from PubMed. It resulted in ~450 articles as of July 2018. In addition, we also included the 98 studies from Liverome (a curated database of liver cancer-related gene signatures) (20). During the initial screening, review articles and the articles lacking relevant information like level of significance (significant *P* value or FDR) were excluded. Finally, the data was systematically curated from 153 studies/articles. We also collected relevant information about Clinical Trials regarding liver cancer biomarker from ClinicalTrials.gov (32).

In CancerLivER, we have systematically compiled comprehensive information about each biomarker or potential biomarker. We named ‘Potential Biomarker’ for those 56 entries in our database, where experiment is performed exclusively only on mice or rats or cell line samples, i.e. where human patient’s cohort samples missing from studies, i.e. 12 studies. The included information regarding biomarker is the type of biomarker (diagnostic/prognostic/predictive), biomolecule, i.e. protein/RNA/miRNA, type of liver cancer, regulation status in the cancerous condition with p-value/FDR, performance measures like sensitivity/specificity/accuracy, patient's cohort used in study for biomarker discovery, publication PMID, publication year, degree of validity, i.e. whether validated on independent dataset or not, clinical trial status with it's NCT number, etc.

### Analysis modules

To incorporate analysis modules, primarily we have pre-processed each dataset matrix individually from each profiling technique for each platform in a standardized manner. For Affymetrix datasets, raw files were pre-processed with background correction and eventually RMA values calculated using the oligo package. For Illumina datasets, raw files were processed using Limma and lumi packages (33,34) and finally log2 values calculated using in-house R scripts. Similarly, raw Agilent one-colour and Agilent two-colour files were pre-processed using Limma package (34) individually, then A-values were generated, which were further transformed to log2 values. Eventually, the average of multiple probes that correspond to a single gene for each dataset individually employing in-house R scripts was computed. To reduce the cross-platform artefacts, quantile normalisation using the preprocessCore package of Bioconductor (35) was performed for each dataset and for each profiling technique. Based on individual techniques, different datasets combined and eventually central tendency measures like mean, median, Q1 (first quartile), Q3 (third quartile), minimum, maximum and standard deviation computed using in-house python and R scripts. Finally, to implement different analysis modules on the web server, we have extracted names/terms and genes lists associated with the KEGG pathway (2016), BioCarta pathways (2016) and GO terms i.e. GO biological processes (2018), GO cellular components (2018) and GO molecular functions (2018) from Enrichr (36). Information associated with genes such as HGNC gene symbol, gene name, Entrez gene ID, Ensembl ID, UniProt IDs, PharmGKB IDs, AltGeneIDs, synonyms and BioGRID IDs obtained using the org.Hs.eg.db package of Bioconductor (37, 38) and from the Comparative Toxicogenomics Database (CTD). To further facilitate the users, a complete Gene_annoation file was provided in ‘Download’ option under ‘Dataset’ and ‘Download’ modules on the web server. Single-sample Gene Set Enrichment Analysis (ssGSEA) has been implemented using GSVA package (39, 40). Further, the Limma package from Bioconductor has been used to compute differentially enriched GO terms or pathways between HCC and non-tumourous samples (29, 34). Highcharts (41) and CanvasJS (42) have been deployed for graphical visualisation of data.

### Database architecture and web interface

**Figure 2:**
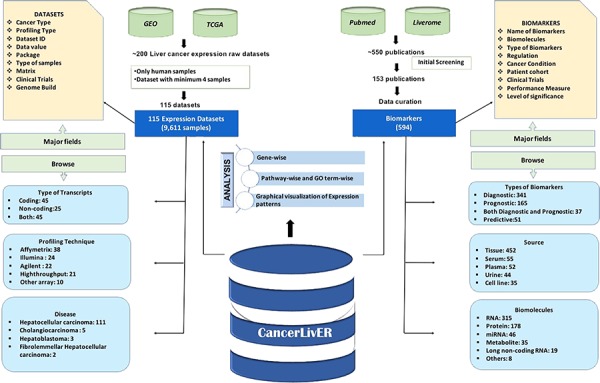
Architecture of CancerLivER.

CancerLivER is built on the Apache HTTP server (version 2.4.7), which is installed on a machine with Ubuntu as an operating system. The responsive front-end, which is suitable for mobiles, tablets and desktops, was developed using HTML5, CSS3, PHP5 and JavaScript. MySQL (a relational database management system, version 5.5.55) was used at the back-end to manage the data. The architecture of CancerLivER is shown in Figure 2.

## Results

### Resource statistics

CancerLivER is a comprehensive resource that contains 115 dataset matrices for expression profiles of 9611 samples and 594 potential biomarkers of liver cancer and extracted from public repositories and literature, respectively.

### Datasets

It contains a total of 115 datasets of expression profiles of liver cancer. There are 111 datasets of HCC, 5 for cholangiocarcinoma, 3 for hepatoblastoma and 2 for fibrolamellar HCC. The major part of the datasets in our database is for HCC which might be due to the fact that HCC is the primary malignancy among liver cancer. Further, due to lack of sufficient data for most of rare liver cancer types like liver lymphoma, hepatic osteogenic sarcoma and liver angiosarcoma, CancerLivER does not contain any dataset for them. CancerLivER contains 38, 24, 22, 21 and 10 datasets generated from Affymetrix array, Illumina array, Agilent array, high-throughput and other profiling techniques, respectively. Based on the type of transcripts, this resource holds 45 datasets of protein coding, 25 of non-coding and 45 datasets of both protein-coding and non-coding types of transcripts. The statistics of datasets present in CancerLivER are shown in Table 1.

**Table 1:** Statistics of the distribution of datasets and biomarkers in CancerLivER

**Distribution of datasets**					
**Based on liver cancer types**		**Based on types of transcripts**		**Based on profiling techniques**	
Hepatocellular carcinoma (HCC)	111	Coding	45	Affymetrix	38
Cholangiocarcinoma (CCA)	5	Non-coding	25	Illumina	24
Hepatoblastoma	3	Both	45	Agilent	22
Fibrolamellar hepatocellular carcinoma	2			High-throughput	21
				Other array	10
**Distribution of biomarkers**					
**Based on type of Biomarkers**		**Based on biomolecule**		**Based on source of samples**	
Diagnostic	341	RNA	315	Tissue	452
Prognostic	165	Protein	178	Serum	55
Predictive	51	miRNA	46	Plasma	52
Both diagnostic and prognostic	37	Metabolite	35	Urine	44
		lincRNA	19	Cell line or cells	35
		Others	8		

### Biomarkers from the literature

The 153 studies harbour 594 entries for potential biomarker candidates of liver cancer. There are 538 entries for Biomarkers and 56 entries for potential Biomarker candidates. We called Biomarker to only those 538 entries from 141 studies, where experimental analysis involves human patient’s cohort samples, whereas we named ‘Potential Biomarker candidate with *’ for the rest of the 56 entries for which information is extracted from those studies (12 studies), where experiment is performed exclusively only on mice or rat or cell line samples and lacks human patient’s cohort samples. The maximum number of reported biomarkers (564 out of 594) is for HCC owing to the most commonly occurring type of liver cancer and hence most sought after. There are only 9 reported biomarkers for cholangiocarcinoma, 3 for hepatoblastoma and 15 for other liver disease conditions. Further, there were 341 entries for diagnostic, 165 for prognostic, 37 for both diagnostic and prognostic and 51 predictive, among 594 entries. Based on Biomolecules, it collocates 315 RNA, 178 protein, 46 miRNA, 35 metabolite, 19 long non-coding RNA and 8 other categories of biomarkers. Here, these biomarkers extracted from major sources like tissue, serum, plasma, urine and cell lines with the numbers corresponding to 452, 55, 52, 44 and 35, respectively. There are a number of studies in literature that elucidated biomarker potential of a specific biomarker or their combination in different cohorts; this resource encompasses the information from all such reports. For instance, there are 24 entries for AFP, a protein biomarker from 13 different studies in our database. The top five genes/proteins that are reported as a constituent of biomarker or signature in at least five different studies are shown in Table 2. Besides, this resource has nearly 500 unique biomarkers or biomarker candidates. Furthermore, CancerLivER linked the 63 entries of biomarkers with the clinical trials for liver cancer. We also provided a field named as ‘Degree of validity’ on our database, where we have added information whether the biomarker is validated on independent patients’ cohort or not, which indicate the robustness of the biomarker. All the statistics regarding biomarker entries in the CancerLivER are given in Table 1.

**Table 2:** List of genes/proteins reported as biomarker/signature in at least five different studies

**Biomarker/gene**	**Biomolecule**	**No. of entries**	**No. of studies**
AFP	Protein	24	13
*GPC3*	RNA	6	6
*IGFBP3*	RNA	6	6
*VIM*	RNA	6	6
*CD24*	RNA	5	5

### Implementation of web tools

A number of tools have been integrated for data retrieval and data analysis; the following is a brief description of different options available in CancerLivER.

### Data retrieval tools

We have incorporated different modules each for Dataset Search and Biomarker Search to facilitate easy retrieval of data. These modules include Keyword Search, Complex Search and various browsing tools.

### Keyword Search

Keyword Search facilitates users to search the dataset and biomarker according to any of the desired query in the Resource. Further, users can also select the desired fields to display in the results.

### Complex Search

In Complex Search, users can execute complex and multiple queries for extracting desired data from the resource. This module allows the use of standard logical operators (‘=’, ‘>’, ‘<’ and ‘LIKE’). A user can combine the outputs of different queries using operators like ‘AND & OR’.

### Browsing tools

In CancerLivER, we have provided a simple yet thorough class-wise browsing facility, in which all the datasets and biomarkers have been categorized into different classes. In this module, information related to the dataset can be browsed using the following three criteria: (i) type of array technique employed, (ii) disease type and (iii) type of transcript; and the information related to a biomarker can be browsed using the following four categories: (i) biomarker or gene, (2) biomarker type, (iii) biomarker biomolecule and (iv) source of biomarkers.

### Analysis tools

This tool of CancerLivER allows the users to analyse and visualize the expression pattern of desired genes in various types of samples like HCC, cholangiocarcinoma, fibrolamellar HCC, hepatoblastoma and normal healthy and adjacent non-tumour, of liver cancer among different datasets which implemented various profiling techniques, i.e. Affymetrix, Agilent and Illumina or TCGA in terms of graphs and box plots. To further facilitates the user, we have incorporated four modules: (i) single-gene-wise, where the user can enter or select the desired gene from list; (ii) multiple-genes-wise, where the user can paste the desired list of genes; (iii) pathway-wise, where the user can select a specific pathway (KEGG or BioCarta pathways) gene and see the status of genes involved in this pathway; and (iv) GO-term-wise, where the user can select a specific GO term (GO biological process (BP) or GO cellular component (CC) or GO molecular function (MF)) genes, to analyse and visualize the expression pattern of his/her desired genes among different types of samples in various types of datasets. In addition, the Multiple Gene Analysis option allows the user to compute the ssGSEA score for GO terms and KEGG pathways. Here, the user can also visualize significant enriched terms or pathways for HCC vs non-tumourous samples.

### Download

This module provides a facility for the user to download any of the datasets as the dataset matrix maintained in the CancerLivER.

### Important links

This tool provides links to all important repositories associated with liver cancer and genomic data.

### Web server availability

CancerLivER is responsive and compatible with all the latest gadgets and can be freely accessed at https://webs.iiitd.edu.in/raghava/cancerliver/.

## Discussion

With the advent of the microarray and RNA-Seq technologies, there is a tremendous increase in the generation of transcriptome data for different types of tumour-associated studies. The integration of multi-dimensional transcriptomic data and its analysis is vital to delineating the comprehensive understanding of tumorigenesis in cancer (43–45). Liver cancer is one of the most lethal malignancies (46). The availability of this huge amount of data opens great opportunities for the analysis of gene expression quantification and identification of stable signature genes associated with liver cancer. The delineation of biological significance from these data is often skewed due to the lack of adequate data matrices in the uniform format for manipulation and analysis. To fill this lacuna, we present CancerLivER, a platform/Resource integrating 115 annotated datasets for liver cancer encompassing expression profiles with clinical information for 9611 samples. Here, a user can query each dataset using various keywords like its profiling technique, type as well as number of samples, type of transcripts present in the data, data values, processing package and genome build. In addition, the user can download complete annotated dataset matrices to perform analysis on multiple datasets.

**Figure 3:**
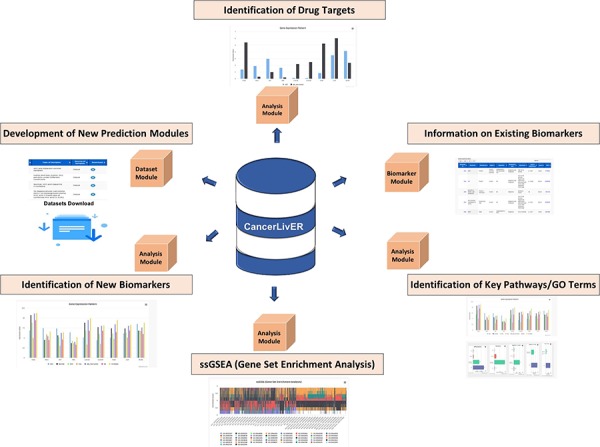
Potential applications of CancerLivER.

**Figure 4:**
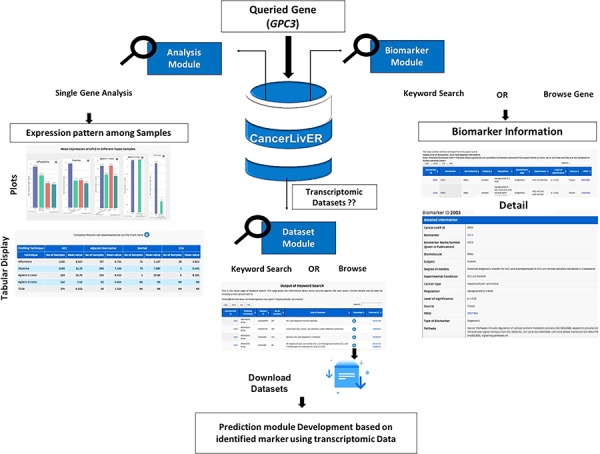
The workflow shows the process of application of CancerLivER in the prioritisation of GPC3 as a diagnostic biomarker and as a drug target for HCC.

Besides, CancerLivER contains 594 entries for biomarkers or potential biomarker candidates for liver cancer. There are 538 entries for Biomarkers and 56 entries for Potential Biomarkers in our database. Recent literature raises the concern for the slow progress in the development of predictive biomarkers for targeted and other novel treatment options (47). Therefore, we have also included the 56 entries for potential biomarker candidates from 12 studies where experiment is performed solely on cell lines or mice or rats. So, researchers can explore the potential of these candidate signatures on human patients’ cohort. It helps to enhance the progress of development of these biomarkers towards their clinical utility. However, there is a liver cancer-related gene signature resource, i.e. Liverome (20), that accommodates data regarding signatures mined from literature up to the Year 2010. It maintains information related to the signature gene, evidence in terms of regulation status (up/down), fold change and *P* value in different disease conditions, its chromosomal location, associated gene ontology terms and the publication from which information extracted. In CancerLivER, we incorporated information from the articles published till July 2018 in addition to articles in the Liverome. Furthermore, we have also included additional information regarding patient cohorts, performance measures, i.e. sensitivity, specificity, accuracy and AUROC, whether the signature can be explored as diagnostic, prognostic or predictive biomarkers, biomarker biomolecule, cancer type, disease condition, regulation status, level of significance (in terms of *P* value or FDR), pathways associated with biomarkers, source of biomarker, PMID, year and clinical trial information, i.e. NCT number regarding biomarker. CancerLivER is freely accessible to the research community to query and analyse transcriptome data for liver cancer. Data retrieval tools include ‘Dataset Search’ and ‘Biomarker Search’ for a simple and complex query. Additionally, ‘Browse’ tools allow the user to query data based on specific category. Furthermore, CancerLivER offers an analysis tool by which a user can analyse and visualize the expression pattern of his desired genes, genes of desired biological pathways such as KEGG and BioCarta and genes associated with desired GO terms like GO biological process, GO cellular component and GO molecular function among different types of samples in various types of datasets.

### Major features of CancerLivER

CancerLivER is a dedicated web portal that encompasses comprehensive information regarding liver cancer-specific biomarkers, store expression data and implemented interactive analysis tools. Eventually, it facilitates convenient retrieval of information for better understanding and management of this lethal malignancy. It offers a variety of applications for the scientific community who have been actively researching in the field of liver cancer like identification of new biomarkers, drug targets, development of prediction models, etc., as shown in Figure 3, owing to its alluring features as given below:

(i) The quality-controlled raw data processed in a standardized manner to generate annotated data matrix (115 data matrices of expression profiles of 9611 samples) for each type of profiling technique with clinical information of samples in a simple csv format.
(ii) Storehouse for 594 liver cancer-associated biomarkers manually curated from literature.
(iii) Simple data query tools for extraction of information regarding biomarkers and datasets from the resource.
(iv) Convenient browsing tools for data retrieval based on specific category.
(v) Tabular and graphical display of information.
(vi) Interactive data analysis and intuitive visualisation tools for gene expression landscape comparison among various types of samples in different types of datasets based on genes, pathway or GO term.
(vii) ssGSEA analysis offers biological implication of queried set of genes.

### Case study

In CancerLivER, the user can easily fetch information regarding biomarkers, expression pattern of genes and datasets for liver cancer based on his query using simple keywords. Subsequently, the user can also generate his hypothesis or design experiment. For instance, if a user wishes to query *GPC3* gene, the user will fetch six entries from six different studies against his query under the ‘Biomarker’ module using either a ‘Keyword Search’ or ‘Browse by Gene’ tools. Here, the user will get the detailed information regarding *GPC3.* This search will show the six entries for *GPC3*, the database reports that this gene has been identified as a potential diagnostic biomarker in five and prognostic biomarker in one study for HCC. The ‘Degree of validity’ shows that this gene has been validated on independent datasets in most of the studies. Yet, this gene is not involved in clinical trials. Further, if one clicks on ‘CancerLiverID’, the database provides detailed information like human tissue sample which is used for experiment. Further, employing the ‘Analysis’ module, the user can also analyse the expression pattern of his desired gene among various types of samples like HCC, non-tumourous/normal and CCA in different datasets. Here, it can be observed that the mean expression of *GPC3* is higher in HCC in comparison to other types of samples in most of the datasets. So, the user can hypothesize whether *GPC3* can be explored as biomarker or drug target or not. Further, users can also enquire what kind of transcriptomic datasets are available in CancerLivER using the ‘Dataset’ module and subsequently can download desired datasets. Eventually, based on his hypothesis, a user can also perform analysis on these datasets and can also develop prediction models or design drug targets employing several bioinformatics approaches. Figure 4 depicts the workflow of how CancerLivER can facilitate researchers regarding their queried gene to prioritize it as disease biomarker and drug target.

In the absence of CancerLivER, a researcher would have to read vast literature in the form of bulky texts scattered in different platforms to make any inferences regarding biomarkers and drug targets. Further, a researcher would have to visit numerous repositories and need to have bioinformatics skills to extract data and to generate a uniformly formatted dataset matrix to perform any analysis or design experiment. The tabular display of information regarding liver cancer biomarkers, the availability of uniformly formatted expression datasets and the interactive analysis modules in CancerLivER makes it convenient for researchers to glance at all the vital information in hand clearly and in a hassle-free manner.

### Update of CancerLivER

CancerLivER will be updated regularly to provide up-to-date information. The current version of CancerLivER contains only RNA expression-related datasets. Besides the expression datasets, other genomic features (e.g. mutations, CNV, epigenomics, proteomics) are equally important. In future, attempt will be made to update this data to provide more comprehensive and up-to-date information.

## Contributions

H.K. manually collected and curated the expression datasets. H.K. and S.B. developed the pipelines to annotate and design the expression dataset matrices. H.K. and D.K. manually collected and curated biomarkers related the data. H.K., S.B. and G.P.S.R analysed the data. H.K. and D.K. developed the web interface. H.K., S.B. and G.P.S.R. prepared the manuscript. G.P.S.R. conceived and coordinated the project, helped in the interpretation and analysis of data, refined the drafted manuscript and gave complete supervision to the project. All of the authors read and approved the final manuscript.

## Data Availability Statement

CancerLivER can be freely accessed at following URL: https://webs.iiitd.edu.in/raghava/cancerliver, and data is available with a CC-BY 4.0 license.

## Supplementary Material

Revised_Manuscript_with_highlighted_changes_baaa012
