# Supplementary material for: CancerLivER: a database of liver cancer gene expression resources and biomarkers
Source: Database (Oxford). 2020 Mar 7;2020:baaa012. doi: 10.1093/database/baaa012 (PMC7061090; doi:10.1093/database/baaa012)
Supplement: Revised_Manuscript_with_highlighted_changes_baaa012 [file revised_manuscript_with_highlighted_changes_baaa012.doc]

**CancerLivER: A Database of Liver Cancer Gene Expression Resources and Biomarkers**

Harpreet Kaur^1,2^, Sherry Bhalla^2,3^, Dilraj Kaur^2^, Gajendra P.S. Raghava^2^

1. CSIR-Institute of Microbial Technology, Chandigarh, India.
2. Department of Computational Biology, Indraprastha Institute of Information Technology, New Delhi, India.
3. Centre for Systems Biology and Bioinformatics, Panjab University, Chandigarh, India.

**Emails of Authors:**

Harpreet Kaur: [harpreet5aug@imtech.res.in](mailto:harpreet5aug@imtech.res.in); hks04180@gmail.com

Sherry Bhalla: [s.sherry308@gmail.com](mailto:s.sherry308@gmail.com)

Dilraj Kaur: [dilrajk@iiitd.ac.in](mailto:dilrajk@iiitd.ac.in)

GPS Raghava: [raghava@iiitd.ac.in](mailto:raghava@iiitd.ac.in)

**^*^CORRESPONDENCE**

Professor, Center for Computational Biology

Indraprastha Institute of Information Technology,

Okhla Industrial Estate, Phase III, New Delhi 110020,

India. Tel.: +91 011 26907444

E-mail address: [raghava@iiitd.ac.in](mailto:raghava@iiitd.ac.in)

**Abstract**

**Background:** Liver cancer is the fourth major lethal malignancy worldwide. To understand the development and progression of liver cancer, biomedical research generated a tremendous amount of transcriptomics and disease-specific biomarkers data. However, dispersed information poses pragmatic hurdles to delineate the significant markers for the disease. Hence, a dedicated resource for liver cancer is required that integrates scattered multiple formatted datasets and information regarding disease-specific biomarkers.

**Result: Liv**er **Cancer** **E**xpression **R**esource (CancerLivER) is a database that maintains gene expression datasets of liver cancer along with the putative biomarkers defined for the same in the literature. It manages 115 datasets that include gene-expression profiles of 9,611 samples. Each of incorporated datasets was manually curated to remove any artifact, subsequently standard and uniform pipeline according to the specific technique is employed for their processing. Additionally, it contains comprehensive information on 594 liver cancer biomarkers include mainly 315 gene-biomarkers or signatures, 178 protein and 46 miRNA-based biomarkers. To explore the full potential of data on liver cancer, a web-based interactive platform developed to perform search, browsing and analyses. Analysis tools also integrated to explore and visualize the expression patterns of desired genes among different types of samples based on individual gene, GO ontology and pathways. Furthermore, dataset matrix download facility provided to facilitate the users for their extensive analysis to elucidate more robust disease-specific signatures. Eventually, CancerLivER is a comprehensive resource which is highly useful for the scientific community working in the field of liver cancer.

**Availability:** CancerLivER can be accessed on the web at <https://webs.iiitd.edu.in/raghava/cancerliver>.

**Keywords**

Resource; Liver Cancer; transcriptomics; Biomarkers; Expression; Datasets

**Introduction**

According to GLOBOCAN 2018, liver cancer is among the top five cancers with the highest mortality rate that accounts for 8.2% of the deaths caused by cancer. Nearly 841,000 new cases and 782,000 deaths of liver cancer have been estimated in 2018 worldwide (1). The major type of liver cancer is hepatocellular carcinoma (HCC) which accounts for nearly 75% to 85% of liver cancer cases. Other types of liver cancer is the cancer of bile duct (cholangiocarcinoma) that accounts for the 10 to 20 % of total cases of liver cancer. Further, Fibrolamellar Carcinoma, Hepatoblastoma, Angiosarcoma and Hemangiosarcoma are among rare types of liver cancer. Fibrolamellar carcinoma tends to develop in adults of 20-30 years old, and it’s not usually associated with cirrhosis or infection with hepatitis B or C, while Hepatoblastoma usually affects young children, i.e. children under three years. Angiosarcoma and Hemangiosarcoma are the other rare types of liver cancers that start in the cell lining the blood vessels of the liver (2).

With the advancement in the field of genomics, enormous data have been generated to study the transcriptome expression of cancer samples to gain insights about the physiology of the disease. In the past, a wide range of high-throughput studies have been performed to identify cancer-specific biomarkers. The data generated from most of these high-throughput studies have been deposited over the time in various databases like Genomic Data Common (GDC) data portal (3), International Cancer Genome Consortium Data Portal (ICGC) (4), The Cancer Genome Atlas (TCGA) (5) and Gene Expression Omnibus (GEO) (6). These databases primarily act as raw data repositories. Therefore, it is a formidable task to ascertain the biological significance from these data and make it available to users with an easy interface to analyse the data. This requires highly developed bioinformatics skills to annotate and generate appropriate data matrices to extract vital genes associated with the disease. Although, GEO contains both raw and processed datasets, however, the processed datasets implement diverse processing and normalization techniques. Therefore, the heterogeneity in the processed data poses major obstacles to compare various transcriptomics datasets. Thus, it is important to employ a uniform pipeline for the processing of the large number of raw datasets generated by a specific technique, i.e. Affymetrix or Illumina, etc. to analyse them in a comprehensive manner to dissect more robust gene signatures for the disease.

In the recent past, there are a number of dedicated genomics, proteomics and peptidomics web-resources and platforms designated for different types of cancers or disease condition like Colorectal Cancer Atlas (7), CRCRpred (8), IGDB-NSCLC (9), CancerPPD (10), Cancertope (11), HCMDB (12), StemMapper (13), CancerCSP (14), CancerPDF (15), CancerSPP (16), PhenoDIS (17), RareLSD (18) and Clinical Genomic Database (CGD) (19) covering various disease conditions including colorectal cancer, non-small cell lung carcinoma, metastatic cancers, renal cell cancer, skin cancer and rare diseases, etc.

There is an enormous generation of information and expression profiles of liver cancer patients which are deposited in various repositories and literature in different formats. Till date, there is no dedicated platform which maintains uniform datasets of liver cancer. Although, there is a resource Liverome (20), a curated database of liver cancer-related gene signatures which harbour information regarding signature genes associated with liver cancer from articles published up to the year 2010. It was not updated after its first publication in the year 2011. Besides, few of expression datasets for liver cancers are also available in resources like cBioPortal (21), BioXpress (22), and OncoMX (23). Among them, cBioportal contains a total of eight datasets for liver cancer; three of which are of expression datasets, while, five are of mutation datasets. BioXpress and OncoMX have taken the expression dataset from TCGA only. In addition, recently web portals such as HCCPred and CancerLSP are also developed for HCC prediction and the stage identification of liver cancer using transcriptomics and epigenomics data implementing machine learning algorithms, respectively (24,25). This indicates that the information and datasets are widely scattered across different resources. Thus, there is a need to develop an integrated dedicated user-friendly public web-resource or platform which catalogues the uniform data matrices for each specific type of expression profiling technique and the information regarding already identified/existing important markers for liver cancer.

To complement the existing database, here we present the liver cancer resource named as CancerLivER (https://webs.iiitd.edu.in/raghava/cancerliver), which provides annotated uniform matrices of published liver cancer expression profiles for each type of expression profiling techniques. Besides expression profiles, this resource also encompasses the information of various types of biomarkers or potential biomarker candidates for liver cancer mined from literature. This database is freely accessible to the research community to query and analyse liver cancer-related expression data and biomarkers.

**Material and Methods**

**Data Collection**

**Gene-expression profiles datasets**

Systematic data searching was conducted for liver cancer expression profiles using the following keywords: ‘Liver cancer’, with customized criteria on study type “Expression profiling by array” , “Expression profiling by high throughput sequencing”, “Non-coding RNA profiling by array” and “Non-coding RNA profiling by high throughput sequencing” and criteria on organism “Homo sapiens” from GEO (6) and GDC data portal (3). The extracted data are limited to human studies published before May 2018. A total of ~200 raw or supplementary datasets were initially downloaded using GEOquery package (26) and gdc-client from GEO and GDC data portal, respectively. Then, we manually curated these datasets to ensure that the data contains only expression data from human samples. Those datasets were excluded from the study where expression data from a) cell lines, b) mice or rat. The number of datasets was reduced to ~160. Further, we exclude those datasets where Superseries are present and whose Sub-Series were already included in the study; and those datasets were also excluded from the study for which raw files were corrupted and where probe ID and HGNC Gene symbols were not available. Subsequently, only those datasets were included, which have enough number of samples (at least 2 pairs, i.e. 4 samples). Eventually, the gene expression profiles of 115 datasets remained, including a total of 9,611 samples that include cancerous, normal adjacent non-tumor, cirrhotic condition, etc. In addition to expression profiles of samples, we also retrieved clinical information for the samples wherever available.

***Processing of datasets***

Each retrieved raw dataset (supplementary data) was subjected to a detailed curation process. For processing of Affymetrix gene expression datasets, the raw data (CEL files) were processed and normalized to RMA values implementing oligo package from the bioconductor (27) using customized R pipeline. For processing of Agilent array data (both 1-color and 2-color), raw files were processed and normalized to A-values implementing Limma package (28,29) from the bioconductor using customized R-pipeline, in which at first 2-color microarrays background was corrected using “normexp” with offset 0. For the processing of Illumina data, raw files were processed implementing Limma (28,29) and lumi packages (30,31) from the bioconductor (29) using customized R-pipeline. High-throughput datasets were manually curated using in-house scripts to make an appropriate matrix from different sample files for each dataset.

The raw records for each dataset were manually inspected to extract relevant information including type, number, the source of samples and clinical information like age, gender, vital status, and survival status, etc. Further, each dataset carefully investigated to check appropriate sample ID, type of sample and to remove any irrelevant error in dataset file. In addition, HGNC Gene symbol and Entrez Gene ID were extracted from respective Platform file (wherever available) and incorporated in the dataset matrix for each dataset. Eventually, a ready-to-use matrix for each dataset was prepared which consisted of clinical information (wherever available) and expression profile of each gene/probe corresponding to each sample. The Figure 1 represents the workflow to generate the final expression profile matrices.

**Insert Figure 1 Here**

***Biomarker data***

The data was manually collected and curated from published research articles. Only those candidate biomarkers were included in the resource, whose biomarker potential was experimentally determined. We queried PubMed to search for research articles regarding biomarkers for liver cancer. The query ‘(Liver cancer [Title/Abstract]) AND Biomarkers [Title/Abstract]’ was used to retrieve the articles relevant to liver cancer biomarkers from PubMed. It resulted in ~450 articles as of July 2018. In addition, we also included the 98 studies from Liverome (a curated database of liver cancer-related gene signatures) (20). During the initial screening, review articles and the articles lacking relevant information like level of significance (significant p-value or FDR) were excluded. Finally, the data was systematically curated from 153 studies/articles. We also collected relevant information about Clinical Trials regarding Liver cancer biomarker from ClinicalTrials.gov (32).

In CancerLivER, we have systematically compiled comprehensive information about each biomarker or potential biomarker. We named “Potential Biomarker” for those 56 entries in our database, where experiment is performed exclusively only on mice or rats or cell line samples, i.e. where human patient’s cohort samples missing from studies, i.e. 12 studies. The information includes the biomarker, biomolecule, i.e. RNA or Protein or miRNA or Lnc-RNA, type of biomarker, i.e. diagnostic, prognostic, predictive, Degree of validity, i.e. whether it is validated or not on independent dataset, liver cancer type, experimental condition, regulation status of biomarker in cancerous condition (including fold change or fisher ratio), level of significance (p-value or FDR), performance measures like sensitivity, specificity, accuracy and AUROC; patients cohort used in the study, PMID, publication year, and information regarding whether biomarker is in clinical trial and its NCT number, etc.

**Analysis modules**

To incorporate analysis modules, primarily we have pre-processed each dataset matrix individually from each profiling technique for each platform in a standardized manner. For Affymetrix datasets, raw files were pre-processed with background correction and eventually RMA values calculated using the oligo package. For Illumina datasets, raw files were processed using Limma and Lumi packages (33,34) and finally log2 values calculated using in-house R scripts. Similarly, raw Agilent-1-color and Agilent-2-color files were pre-processed using Limma package (34) individually, then A-values were generated, which were further transformed to log2 values. Eventually, the average of multiple probes computed that correspond to a single gene for each dataset individually employing in-house R scripts. To reduce the cross-platform artifacts, quantile normalisation using the PreprocessCore package of Bioconductor (35) is performed for each dataset and for each profiling technique. Based on individual technique, different datasets combined and eventually central tendency measures like mean, median, Q1 (first quartile), Q3 (third quartile), minimum, maximum, standard deviation computed using in-house python and R scripts. Finally, to implement different analysis modules on webserver, we have extracted names/terms and genes lists associated with KEGG pathway (2016), biocarta pathways (2016), GO terms i.e. GO Biological Processes (2018), GO cellular Components (2018) and GO Molecular functions (2018) from Enrichr (36). Information associated with Gene like HGNC Gene symbol, Gene Name, Entrez Gene ID, Ensembl ID, UniProtIDs, PharmGKBIDs, AltGeneIDs, Synonyms, BioGRIDIDs, etc. obtained using org.Hs.eg.db package of Bioconductor (37,38) and from The Comparative Toxicogenomics Database (CTD). To further facilitate the users a complete Gene_annoation file provided in “Download” option under “Dataset” and “Download” modules on the webserver. Single sample Gene Set Enrichment Analysis (ssGSEA) has been implemented using GSVA package (39,40). Further, Limma package from Bioconductor has been used to compute differentially enriched GO terms or pathways between HCC and non-tumorus samples (29,34). Highcharts (41) and CanvasJS (42) has been deployed for graphical visualisation of data.

**Database Architecture and Web Interface**

CancerLivER is built on Apache HTTP server (version 2.4.7), which is installed on a machine with Ubuntu as an operating system. The responsive front-end, which is suitable for mobile, tablet and desktop, was developed using HTML5, CSS3, PHP5, and JavaScript. MySQL (a relational database management system, version 5.5.55) was used at the back-end to manage the data. The architecture of CancerLivER is shown in Figure 2.

**Insert Figure 2 Here**

**Results**

**Resource Statistics**

CancerLivER is a comprehensive resource that contains 115 dataset matrices for expression profiles of 9,611 samples and 594 potential biomarkers of liver cancer and extracted from public repositories and literature, respectively.

**Datasets**

It contains a total of 115 datasets of expression profiles of liver cancer. There are 111 datasets of Hepatocellular carcinoma, 5 for Cholangiocarcinoma, 3 for Hepatoblastoma and 2 for fibrolamellar hepatocellular carcinoma. Major part of the datasets in our database is for HCC that might be due to the fact that HCC is the primary malignancy among liver cancer. Further, due to lack of sufficient data for most of rare liver cancer types like liver lymphoma, hepatic osteogenic sarcoma and liver angiosarcoma, CancerLivER doesn’t contains any dataset for them. CancerLivER contains 38, 24, 22, 21 and 10 datasets generated from Affymetrix array, Illumina array, Agilent array, High-throughput and other profiling techniques, respectively. Based on the type of transcripts, this resource holds 45 datasets of Protein coding, 25 of non-coding and 45 datasets of both protein-coding and non-coding types of transcripts. The statistics of datasets present in CancerLivER are shown in Table 1.

**Insert Table 1 Here**

**Biomarkers from the literature**

The 153 studies harbour 594 entries for potential biomarker candidates of liver cancer. There are 538 entries for Biomarkers and 56 entries for Potential Biomarker candidates. We called biomarker to only those 538 entries from 141 studies, where experimental analysis involve human patient’s cohort samples. Whereas, we named “Potential Biomarker candidate with *” for rest 56 entries for which information is extracted from those studies (12 studies), where experiment is performed exclusively only on mice or rat or cell line samples and lacks human patient’s cohort samples. The maximum number of reported biomarkers (564 out of 594) is for Hepatocellular carcinoma owing to the most commonly occurring type of liver cancer and hence most sought after. There are only 9 reported biomarkers for cholangiocarcinoma, 3 for hepatoblastoma and 15 for other liver disease conditions. Further, 341 entries for diagnostic, 165 prognostic, 37 are both diagnostic as well as prognostic and 51 predictive, among 594 entries. Based on biomolecules, it collocates 315 RNA, 178 protein, 46 miRNA, 35 metabolites, 19 Long non-coding RNA and 8 other categories of biomarkers. Here, these biomarkers extracted from major sources like tissue, serum, plasma, urine and cell lines with the number corresponding to 452, 55, 52, 44 and 35, respectively. There are a number of studies in literature, which elucidated biomarker potential of a specific biomarker or their combination in different cohorts, this resource encompasses the information from all such reports. For instance, there are 24 entries for AFP, a protein biomarker from 13 different studies in our database. Top 5 genes/proteins that are reported as a constituent of biomarker or signature in at least 5 different studies enlisted in Table 2. Besides, this resource has nearly 500 unique biomarkers or biomarker candidates. Furthermore, CancerLivER linked the 63 entries of biomarkers with the clinical trials for liver cancer. We also provided a field named as “Degree of validity” on our database, where we have added information whether biomarker is validated on independent patients ‘cohort or not, which indicate the robustness of biomarker. All the statistics regarding biomarker entries in the CancerlivER given in Table 1.

**Insert Table 2 Here**

**Implementation of Web Tools**

A number of tools have been integrated for data retrieval and data analysis; following is the brief description of different options available in CancerLivER.

**Data retrieval tools**

We have incorporated different modules each for Dataset Search and Biomarker Search to facilitate easy retrieval of data. These modules include Keyword search, Complex search and various browsing tools.

**Keyword Search**

Keyword Search facilitates users to search dataset and biomarker according to any of the desired query in the Resource. Further, users can also select the desired fields to display in the results.

**Complex Search**

In Complex Search, users can execute complex and multiple queries for extracting desired data from the resource. This module allows the use of standard logical operators (“=”, “>”, “<” and “LIKE”). A user can combine the outputs of different queries using operators like “AND & OR”.

**Browsing Tools**

In CancerLivER, we have provided a simple yet thorough class-wise browsing facility, in which all the dataset and biomarker have been categorized into different classes. In this module, the information related to dataset can be browsed using the following 3 criteria (1) Type of array technique employed, (2) Disease type, (3) Type of transcript; and the information related to a biomarker can be browsed using the following 4 categories: (1) Biomarker or Gene, (2) Biomarker type, (3) Biomarker biomolecule and (4) Source of biomarkers.

**Analysis Tools**

This tool of CancerLivER allows the users to analyse and visualize the expression pattern of desired genes in various types of samples like HCC, cholangiocarcinoma, Fibrolamellar hepatocellular carcinoma, hepatoblastoma, normal healthy and adjacent non-tumor, etc*.* of Liver cancer among different datasets implemented various profiling techniques, i.e. Affymetrix, Agilent and Illumina or TCGA in terms of graphs and boxplots. To further facilitates the user, we have incorporated four modules: 1) Single Gene-wise, where user can enter or select the desired gene from list; 2) Multiple Genes-wise, where user can paste the desired list of genes; 3) Pathway-wise, where user can select a specific pathway (KEGG or BioCarta pathways) genes and see the status of genes involved in this pathway 3) GO term-wise, where user can select a specific GO term (GO Biological Process (BP) or GO Cellular component (CC) or GO Molecular function (MF)) genes; to analyse and visualize the expression pattern of his/her desired genes among different types of samples in various types of datasets. In addition, Multiple Genes Analysis option allows user to compute the ssGSEA score for GO terms and KEGG pathways. Here, user can also visualize significant enriched terms or pathways for HCC vs non-tumorus samples.

**Download**

This module provides a facility for the user to download any of the datasets as dataset matrix maintained in the CancerLivER.

**Important links**

This tool provides links to all important repositories associated with Liver Cancer and genomic data.

**Webserver Availability**

CancerLivER is responsive and compatible with all the latest gadgets and can be freely accessed at: **https://webs.iiitd.edu.in/raghava/cancerliver/**

**Discussion**

With the advent of the microarray and RNA-seq technologies, there is tremendous increase in the generation of transcriptome data for different types of tumor-associated studies. The integration of multi-dimensional transcriptomic data and its analysis is vital to delineate the comprehensive understanding of tumorigenesis in cancer (43–45). Liver cancer is one of the most lethal malignancies (46). The availability of this huge amount of data opens great opportunities for the analysis of gene expression quantification and identification of stable signature genes associated with liver cancer. The delineation of biological significance from these data is often skewed due to the lack of adequate data matrices in the uniform format for manipulation and analysis. To fill this lacuna, we present CancerLivER, a platform/Resource integrating 115 annotated datasets for liver cancer encompassing expression profiles with clinical information for 9,611 samples. Here, a user can query each dataset using various keywords like its profiling technique, type as well as number of samples, type of transcripts present in the data, data values, processing package, and genome build etc. In addition, the user can download complete annotated dataset matrices to perform analysis on multiple datasets.

Besides, CancerLivER contains 594 entries for biomarkers or potential biomarker candidates for liver cancer. There are 538 entries for Biomarkers and 56 entries for Potential Biomarkers in our database. Recent literature raise the concern for the slow progress in the development of predictive biomarker for targeted and other novel treatment options (47). Therefore, we have also included the 56 entries for potential biomarker candidates from 12 studies where experiment is performed solely on cell lines or mice or rat etc. So, researchers can explore the potential of these candidate signatures on human patients’ cohort. It helps to enhance the progress of development of these biomarker towards their clinical utility. Though, there is a liver cancer-related gene signatures resource, i.e. Liverome (20), that accommodates data regarding signatures mined from literature up to the year 2010. It maintains information related to the signature gene, evidence in terms of regulation status (up/down), fold change and p-value in different disease conditions, its chromosomal location, associated gene ontology terms, the publication from which information extracted. In the CancerLivER, we incorporated information from the articles published till July 2018 in addition to articles in the Liverome. Furthermore, we have also included the additional information regarding patients cohorts, performance measures, i.e. sensitivity, specificity, accuracy and AUROC, whether the signature can be explored as diagnostic, prognostic or predictive biomarkers, biomarker biomolecule, cancer type, disease condition, regulation status, level of significance (in terms of p-value or FDR), pathways associated with biomarkers, source of biomarker, PMID, year, the clinical trial information, i.e. NCT number regarding biomarker. CancerLivER is freely accessible to the research community to query and analyse transcriptome data for liver cancer. Data retrieval tools include ‘Dataset Search’ and ‘Biomarker Search’ for a simple and complex query. Additionally, ‘Browse’ tools allow the user to queried data based on specific category. Furthermore, CancerLivER offers an analysis tool by which a user can analyse and visualize the expression pattern of his desired genes, genes of desired biological pathway such as KEGG and Biocarta and genes associated with desired GO term like GO biological process, GO cellular component and GO Molecular function among different types of samples in various types of datasets.

**Major Features of CancerLivER**

CancerLivER is a dedicated web portal that encompass comprehensive information regarding liver cancer-specific biomarkers, stores expression data and implemented interactive analysis tools. Eventually, it facilitates convenient retrieval of information for better understanding and management of this lethal malignancy. It offers a variety of applications for the scientific community who have been actively researching in the field of liver cancer like identification of new biomarkers, drug targets, development of prediction models, etc. as shown in Figure 3 owing to its alluring features as given below:

1. The quality controlled raw data processed in a standardized manner to generate annotated data matrix (115 data matrices of expression profiles of 9,611samples) for each type of profiling technique with clinical information of samples in simple csv format.
2. Storehouse for 594 liver cancer associated biomarkers manually curated from literature.
3. Simple data query tools for extraction of information regarding biomarkers and datasets from the resource.
4. Convenient browsing tools for data retrieval based on specific category.
5. Tabular and graphical display of information.
6. Interactive data analysis and intuitive visualisation tools for gene expression landscape comparison among various types of samples in different types of datasets based on genes, pathway or GO term.
7. ssGSEA analysis offers biological implication of queried set of genes.

**Insert Figure 3 Here**

**Case Study**

In the CancerLivER, the user can easily fetch information regarding biomarkers, expression pattern of genes and datasets for liver cancer based on his query using simple keywords. Subsequently, user can also generates his hypothesis or design experiment. For instance, if a user wishes to query *GPC3* gene, the user will fetch six entries from six different studies against his query under the “Biomarker” module using either a “Keyword Search” or “Browse by Gene” tools. Here, the user will get the detailed information regarding *GPC3.* This search will show the six entries for *GPC3*, the database reports that this gene has been identified as a potential diagnostic biomarker in five and prognostic biomarker in one study for HCC; The “Degree of validity” shows that this gene has been validated on independent datasets in most of the studies. Yet this gene is not involved in clinical trials.  Further if click on “CancerLiverID”, the database provides detail information like human tissue sample is used for experiment. Further, employing “Analysis” module user can also analyse the expression pattern of his desired gene among various types of samples like HCC, non-tumorous/normal, CCA, etc. in different datasets. Here, it can be observed that the mean expression of *GPC3* is higher in HCC in comparison to other types of samples in most of the datasets. So, the user can hypothesized whether *GPC3* can be explored as Biomarker or Drug target or not. Further, users can also enquire what kind of transcriptomic datasets available in CancerLivER using “Dataset” Module and subsequently can download desired datasets. Eventually, based on his hypothesis, a user can also perform analysis on these datasets and can also develop prediction models or design drug targets employing several bioinformatics approaches.  The Figure 4 depicts the workflow of how CancerLivER can facilitates researchers regarding their queried gene to prioritize it as disease biomarker and drug target.

In the absence of CancerLivER, a researcher would have to read vast literature in the form of bulky texts scattered in different platforms to make any inferences regarding biomarkers and drug targets. Further, a researcher would have to visit numerous repositories and needs to have bioinformatics skills to extract data and to generate a uniformly formatted dataset matrix to perform any analysis or design experiment. The tabular display of information regarding liver cancer biomarkers, the availability of uniformly formatted expression datasets and the interactive analysis modules in CancerLivER makes it convenient for researchers to glance at all the vital information in hand clearly and in a hassle-free manner.

**Insert Figure 4 Here**

**Update of CancerLivER**

CancerLivER will be updated regularly to provide up-to-date information. The current version of CancerLivER contains only RNA expression related datasets. Besides, the expression datasets, other genomics features (e.g., mutations, CNV, epigenomics, proteomics) are equally important. In future, attempt will be made to update this data to provide more comprehensive and up-to-date information.

**Contributions**

H.K. manually collected and curated the expression datasets. H.K. and S.B. developed the pipelines to annotate and design the expression dataset matrices. H.K. and D.K. manually collected and curated biomarkers related the data. H.K., S.B., and G.P.S.R analysed the data. H.K. and D.K. developed the web interface. H.K., S.B. and G.P.S.R. prepared the manuscript. G.P.S.R. conceived and coordinated the project, helped in the interpretation and analysis of data, refined the drafted manuscript and gave complete supervision to the project. All of the authors read and approved the final manuscript.

**Funding**

This research was funded by J. C. Bose National Fellowship (with Grant No. SRP076), Department of Science and Technology (DST), India.

**Acknowledgement**

The authors acknowledge funding agencies J. C. Bose National Fellowship, DST, INDIA. H.K., S.B., and D.K. are thankful to Council of Scientific and Industrial Research (CSIR) India, Indian Council of Medical Research (ICMR), India and IIIT Delhi for providing fellowships.

**Conflicts of Interest**

The authors declare no financial and non-financial conflict of interest.

**Data Availability Statement**

CancerLivER can be freely accessed at following URL: <https://webs.iiitd.edu.in/raghava/cancerliver> and data is available with a CC-BY 4.0 license**.**

**References**

1. New Global Cancer Data: GLOBOCAN 2018 | UICC https://www.uicc.org/new-global-cancer-data-globocan-2018 (accessed Mar 13, 2019).

2. Types | Liver cancer | Cancer Research UK https://www.cancerresearchuk.org/about-cancer/liver-cancer/types (accessed Mar 13, 2019).

3. Grossman, R. L., Heath, A. P., Ferretti, V., et al. (2016) Toward a Shared Vision for Cancer Genomic Data., *N. Engl. J. Med.*, **375**, 1109–12.

4. Zhang, J., Baran, J., Cros, A., et al. (2011) International cancer genome consortium data portal-a one-stop shop for cancer genomics data, *Database*, **2011**.

5. Weinstein, J. N., Collisson, E. A., Mills, G. B., et al. The cancer genome atlas pan-cancer analysis project. *Nat. Genet.* **2013**, *45*, 1113–1120.

6. Barrett, T., Wilhite, S. E., Ledoux, P., et al. (2012) NCBI GEO: archive for functional genomics data sets—update, *Nucleic Acids Res.*, **41**, D991–D995.

7. Chisanga, D., Keerthikumar, S., Pathan, M., et al. (2016) Colorectal cancer atlas: An integrative resource for genomic and proteomic annotations from colorectal cancer cell lines and tissues., *Nucleic Acids Res.*, **44**, D969-74.

8. Lathwal, A., Arora, C. and Raghava, G. P. S. (2019) Prediction of risk scores for colorectal cancer patients from the concentration of proteins involved in mitochondrial apoptotic pathway, *PLoS One*, **14**, e0217527.

9. Kao, S., Shiau, C.-K., Gu, D.-L., et al. (2012) IGDB.NSCLC: integrated genomic database of non-small cell lung cancer., *Nucleic Acids Res.*, **40**, D972-7.

10. Tyagi, A., Tuknait, A., Anand, P., et al. (2015) CancerPPD: a database of anticancer peptides and proteins., *Nucleic Acids Res.*, **43**, D837-43.

11. Gupta, S., Chaudhary, K., Dhanda, S. K., et al. (2016) A Platform for Designing Genome-Based Personalized Immunotherapy or Vaccine against Cancer., *PLoS One*, **11**, e0166372.

12. Zheng, G., Ma, Y., Zou, Y., et al. (2018) HCMDB: the human cancer metastasis database., *Nucleic Acids Res.*, **46**, D950–D955.

13. Pinto, J. P., Machado, R. S. R., Magno, R., et al. (2018) StemMapper: a curated gene expression database for stem cell lineage analysis., *Nucleic Acids Res.*, **46**, D788–D793.

14. Bhalla, S., Chaudhary, K., Kumar, R., et al. (2017) Gene expression-based biomarkers for discriminating early and late stage of clear cell renal cancer., *Sci. Rep.*, **7**, 44997.

15. Bhalla, S., Verma, R., Kaur, H., et al. (2017) CancerPDF: A repository of cancer-associated peptidome found in human biofluids., *Sci. Rep.*, **7**, 1511.

16. Bhalla, S., Kaur, H., Dhall, A., et al. (2019) Prediction and Analysis of Skin Cancer Progression using Genomics Profiles of Patients, *Sci. Rep.*, **9**.

17. Adler, A., Kirchmeier, P., Reinhard, J., et al. (2018) PhenoDis: a comprehensive database for phenotypic characterization of rare cardiac diseases, *Orphanet J. Rare Dis.*, **13**, 22.

18. Akhter, S., Kaur, H., Agrawal, P., et al. (2019) RareLSD: a manually curated database of lysosomal enzymes associated with rare diseases, *Database*, **2019**.

19. Solomon, B. D., Nguyen, A. D., Bear, K. A., et al. (2013) Clinical genomic database, *Proc. Natl. Acad. Sci. U. S. A.*, **110**, 9851–9855.

20. Lee, L., Wang, K., Li, G., et al. (2011) Liverome: a curated database of liver cancer-related gene signatures with self-contained context information., *BMC Genomics*, **12 Suppl 3**, S3.

21. Cerami, E., Gao, J., Dogrusoz, U., et al. (2012) The cBio Cancer Genomics Portal: An open platform for exploring multidimensional cancer genomics data, *Cancer Discov.*, **2**, 401–404.

22. Wan, Q., Dingerdissen, H., Fan, Y., et al. (2015) BioXpress: An integrated RNA-seq-derived gene expression database for pan-cancer analysis, *Database*, **2015**.

23. OncoMX https://www.oncomx.org/ (accessed Dec 13, 2019).

24. Kaur, H., Dhall, A., Kumar, R., et al. (2019) Identification of Platform-Independent Diagnostic Biomarker Panel for Hepatocellular Carcinoma using Large-scale Transcriptomics Data, *Front. Genet.*, **10**, 1306.

25. Kaur, H., Bhalla, S. and Raghava, G. P. S. (2019) Classification of early and late stage liver hepatocellular carcinoma patients from their genomics and epigenomics profiles, *PLoS One*, **14**.

26. Bioconductor - GEOquery http://bioconductor.org/packages/release/bioc/html/GEOquery.html (accessed Mar 14, 2019).

27. Carvalho, B. S. and Irizarry, R. A. (2010) A framework for oligonucleotide microarray preprocessing, *Bioinformatics*, **26**, 2363–2367.

28. Ritchie, M. E., Phipson, B., Wu, D., et al. (2015) limma powers differential expression analyses for RNA-sequencing and microarray studies., *Nucleic Acids Res.*, **43**, e47.

29. Bioconductor - limma http://www.bioconductor.org/packages/release/bioc/html/limma.html (accessed Mar 13, 2019).

30. Du, P., Kibbe, W. A. and Lin, S. M. (2008) lumi: a pipeline for processing Illumina microarray., *Bioinformatics*, **24**, 1547–8.

31. Bioconductor - lumi https://www.bioconductor.org/packages/release/bioc/html/lumi.html (accessed Dec 14, 2019).

32. Home - ClinicalTrials.gov https://clinicaltrials.gov/ (accessed Mar 13, 2019).

33. Du, P., Kibbe, W. A. and Lin, S. M. (2008) lumi: A pipeline for processing Illumina microarray, *Bioinformatics*, **24**, 1547–1548.

34. Ritchie, M. E., Phipson, B., Wu, D., et al. (2015) limma powers differential expression analyses for RNA-sequencing and microarray studies, *Nucleic Acids Res.*, **43**, e47–e47.

35. Bioconductor - preprocessCore https://bioconductor.org/packages/release/bioc/html/preprocessCore.html (accessed Mar 13, 2019).

36. Kuleshov, M. V, Jones, M. R., Rouillard, A. D., et al. (2016) Enrichr: a comprehensive gene set enrichment analysis web server 2016 update., *Nucleic Acids Res.*, **44**, W90-7.

37. Carlson, M. org.Hs.eg.db: Genome wide annotation for Human. R package version 340: Rpackage version 3.4.0 http://bioconductor.org/packages/release/data/annotation/html/org.Hs.eg.db.html (accessed Jan 17, 2020).

38. Davis, A. P., Grondin, C. J., Johnson, R. J., et al. (2019) The Comparative Toxicogenomics Database: Update 2019, *Nucleic Acids Res.*, **47**, D948–D954.

39. Hänzelmann, S., Castelo, R. and Guinney, J. (2013) GSVA: Gene set variation analysis for microarray and RNA-Seq data, *BMC Bioinformatics*, **14**.

40. Bioconductor - GSVA https://www.bioconductor.org/packages/release/bioc/html/GSVA.html (accessed Dec 14, 2019).

41. GitHub - highcharts/highcharts: Highcharts JS, the JavaScript charting framework https://github.com/highcharts/highcharts (accessed Dec 13, 2019).

42. Beautiful HTML5 JavaScript Charts | CanvasJS https://canvasjs.com/ (accessed Mar 13, 2019).

43. Gallo Cantafio, M. E., Grillone, K., Caracciolo, D., et al. (2018) From Single Level Analysis to Multi-Omics Integrative Approaches: A Powerful Strategy towards the Precision Oncology., *High-throughput*, **7**, 33.

44. Sung, J., Kim, P.-J., Ma, S., et al. (2013) Multi-study integration of brain cancer transcriptomes reveals organ-level molecular signatures., *PLoS Comput. Biol.*, **9**, e1003148.

45. Domany, E. (2014) Using high-throughput transcriptomic data for prognosis: a critical overview and perspectives., *Cancer Res.*, **74**, 4612–21.

46. Siegel, R. L., Miller, K. D. and Jemal, A. (2019) Cancer statistics, 2019, *CA. Cancer J. Clin.*, **69**, 7–34.

47. Ocker, M. (2018) Biomarkers for hepatocellular carcinoma: What’s new on the horizon?, *World J. Gastroenterol.*, **24**, 3974.

**Figure legends**

Figure 1: Workflow to generate Expression Dataset matrices maintained in the CancerLivER.

Figure 2: Architecture of the CancerLivER.

Figure 3: Potential Applications of the CancerLivER.

Figure 4: The Workflow shows process of Application of the CancerLivER in the prioritization of GPC3 as a Diagnostic Biomarker and as a Drug Target for HCC.
